# Human Mobility Networks, Travel Restrictions, and the Global Spread of 2009 H1N1 Pandemic

**DOI:** 10.1371/journal.pone.0016591

**Published:** 2011-01-31

**Authors:** Paolo Bajardi, Chiara Poletto, Jose J. Ramasco, Michele Tizzoni, Vittoria Colizza, Alessandro Vespignani

**Affiliations:** 1 Computational Epidemiology Laboratory, Institute for Scientific Interchange (ISI), Torino, Italy; 2 Centre de Physique Théorique (CNRS UMR 6207), Marseille, France; 3 Instituto de Física Interdisciplinar y Sistemas Complejos IFISC (CSIC-UIB), Palma de Mallorca, Spain; 4 Scuola di Dottorato, Politecnico di Torino, Torino, Italy; 5 INSERM, U707, Paris, France; 6 UPMC Université Paris 06, Faculté de Médecine Pierre et Marie Curie, UMR S 707, Paris, France; 7 Complex Systems Lagrange Laboratory, Institute for Scientific Interchange (ISI), Torino, Italy; 8 Center for Complex Networks and Systems Research (CNetS), School of Informatics and Computing, Indiana University, Bloomington, Indiana, United States of America; 9 Pervasive Technology Institute, Indiana University, Bloomington, Indiana, United States of America; 10 Institute for Scientific Interchange (ISI), Torino, Italy; University of Maribor, Slovenia

## Abstract

After the emergence of the H1N1 influenza in 2009, some countries responded with travel-related controls during the early stage of the outbreak in an attempt to contain or slow down its international spread. These controls along with self-imposed travel limitations contributed to a decline of about 40% in international air traffic to/from Mexico following the international alert. However, no containment was achieved by such restrictions and the virus was able to reach pandemic proportions in a short time. When gauging the value and efficacy of mobility and travel restrictions it is crucial to rely on epidemic models that integrate the wide range of features characterizing human mobility and the many options available to public health organizations for responding to a pandemic. Here we present a comprehensive computational and theoretical study of the role of travel restrictions in halting and delaying pandemics by using a model that explicitly integrates air travel and short-range mobility data with high-resolution demographic data across the world and that is validated by the accumulation of data from the 2009 H1N1 pandemic. We explore alternative scenarios for the 2009 H1N1 pandemic by assessing the potential impact of mobility restrictions that vary with respect to their magnitude and their position in the pandemic timeline. We provide a quantitative discussion of the delay obtained by different mobility restrictions and the likelihood of containing outbreaks of infectious diseases at their source, confirming the limited value and feasibility of international travel restrictions. These results are rationalized in the theoretical framework characterizing the invasion dynamics of the epidemics at the metapopulation level.

## Introduction

The human mobility flows that determine the spreading of infectious diseases and the control measures based on limiting or constraining human mobility are considered in the contingency planning of several countries [1]. The target of these control measures is the decrease of travel to/from the areas affected by the epidemic outbreak and the corresponding decline of infected individuals reaching countries not yet affected by the epidemic. While the effects of slowing down the international propagation of an epidemic can be statistically evaluated based on available data and bootstrap techniques [2], the impossibility of disentangling the role played by travel from other contributing factors in the spread of an epidemic [3] has generated discussion about the appropriate strategy for mobility restrictions. In this context the only way to systematically gauge uncertainty and the effectiveness of competing control strategies is through data-driven modeling efforts [4]–[9]. Unfortunately, most previous works have focused on synthetic pandemic influenza scenarios and only a few empirical examples are available to validate models and evaluate the effectiveness of travel restrictions in general [10]–[12].

In the recent 2009 H1N1 pandemic (H1N1pdm), control measures included travel bans to/from Mexico, the screening of travelers on entry into airports, and travel advisories against non-essential travel to Mexico [1]. The aggregation of data on the H1N1pdm therefore represents an unprecedented opportunity to calibrate and validate a modeling approach to the global spread of epidemics that integrates detailed information on human mobility and travel. In the present work, we use the Global Epidemic and Mobility model (GLEaM) [13] that, fully integrating high resolution demographic and mobility data, allows the calibration to the H1N1pdm data of the invasion during the early stage of the epidemic and the exploration of hypothetical scenarios in which reductions in the international travel to/from Mexico with different timing and magnitude are considered. Interventions acting on mobility are found to be scarcely efficient in delaying the invasion process of the pandemic. This computational evidence can be explained within a simplified theoretical framework in terms of a phase transition between invasion and non-invasion dynamics of the metapopulation system, where the critical value is crucially affected by the topological fluctuations of the mobility network.

## Methods

### Model description

The Global Epidemic and Mobility model is based on a metapopulation scheme [4], [8], [9], [14]–[20] in which the world is divided into geographical regions defining a subpopulation network where connections among subpopulations represent the individual fluxes due to the transportation and mobility infrastructure. GLEaM is composed of three different layers [13]: (i) the population layer that integrates census areas for a total of 3,362 subpopulations around major transportation hubs in 220 countries of the world with a resolution up to ¼°×¼° [21]; (ii) the human mobility layer that integrates both commuting flows collected from various sources in more than 30 countries and airline traffic flows provided by the International Air Transport Association (IATA) database [22]; and (iii) the disease dynamics layer that implements a refined *SEIR*-like model [23] taking into account the specific etiology of the H1N1pdm [18].

**Figure 1:**
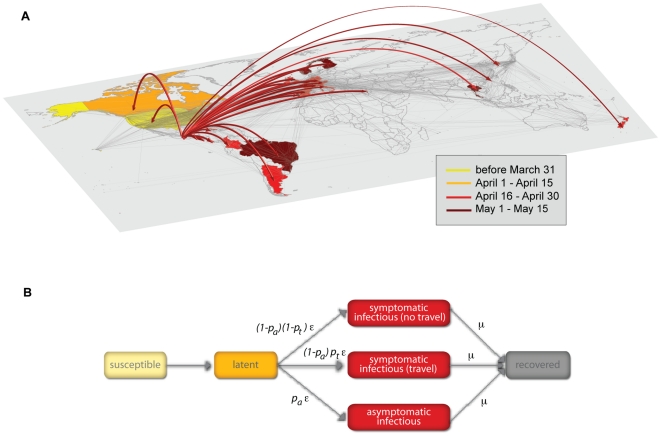
Modeling the 2009 H1N1 pandemic spread with GLEaM. **A**, Illustration of the global invasion of the 2009 H1N1 pandemic during the early stage of the outbreak. The arrows represent the seeding of unaffected countries due to infected individuals traveling from Mexico. The color code indicates the time of the seeding. The map shows the layer of the worldwide air transportation network, which is incorporated into GLEaM. **B**, Compartmental structure in each subpopulation of GLEaM. Each individual is classified by one of the following discrete states: susceptible, latent, symptomatic infectious who can travel, symptomatic infectious who are hampered in their travels by the severity of the illness, asymptomatic infectious, and permanently recovered/removed [23], [26]. We assume that the latency period is equivalent to the incubation period and that no secondary transmissions occur during the incubation period. In addition, the asymptomatic individuals are assumed to be less infectious with respect to the symptomatic ones, with a relative infectiousness *r_β_*, that is half the infectiousness of symptomatic individuals. All parameter values are reported in Table 3 of the SI.

The model simulates short-range mobility between subpopulations with a time scale separation approach that defines the effective force of infections in connected subpopulations [13], [18], [24], [25]. The airline mobility from one subpopulation to another is modeled by an individual based stochastic procedure in which the number of passengers of each compartment traveling from a subpopulation *j* to a subpopulation *l* is an integer random variable defined by the actual data from the airline transportation database [8]. The infection dynamics takes place within each subpopulation. We adopt a *SEIR*-like model [23] in which we consider separate compartments for symptomatic traveling and not traveling, as well as asymptomatic individuals in each subpopulation. More in detail, a susceptible individual in contact with a symptomatic or asymptomatic infectious person contracts the infection at rate *β* or *r_β_β* [26], [27], respectively, and enters the latent compartment where he is infected but not yet infectious. After an average latency period *ε* ^−1^, each latent individual becomes infectious, entering the symptomatic compartments with probability 1−*p*_a_ or becoming asymptomatic with probability *p*_a_ [26], [27]. The symptomatic cases are further divided between those who are allowed to travel (with probability *p*_t_) and those who would stop traveling when ill (with probability 1−*p*_t_) [26]. Infectious individuals recover permanently with rate *µ*. A schematic representation of the compartmental structure is reported in Figure 1. All transitions defining infection dynamics and mobility processes are modeled through binomial and multinomial stochastic variables to mimic the discrete and stochastic nature of the epidemic spreading [8], [13] that is extremely relevant especially at the start of the outbreak (see the SI for details). The time resolution of both mobility and infection dynamics is of one day. Seasonal effects are taken into account by applying a sinusoidal rescaling of the reproductive number according to the time of the year and the hemisphere of location of the subpopulation [4]. In particular, the scaling factor ranges from α_min_ during the summer season to α_max_ during the winter season. Here we consider α_max_ = 1.1, whereas α_min_ assumes the best estimate value obtained from the calibration of the model to the H1N1pdm invasion data (see next Subsection) [18].

### Model calibration

The model is calibrated on the H1N1pdm data. The initial conditions of the epidemic are set near La Gloria, Mexico, on 18 February 2009 in agreement with the information published in official reports and with previous works [18], [28], [29]. Infection parameters describing the transmission potential and the duration of the stages of the disease are obtained through a maximum likelihood procedure based on the empirical data of the H1N1 international seeding events (see Figure 1A). In particular, we use the reproductive number *R_0_* = 1.75 with the generation interval set to 3.6 days (average latency period *ε* ^−1^ = 1.1 days and average infectious period *µ* ^−1^ = 2.5 days). Through a maximum likelihood approach, the above estimates are obtained that best reproduce the actual chronology of newly infected countries (additional details can be found in Ref. [18]). The estimation method is computationally intensive as it involves a Monte Carlo generation of the distribution of arrival time of the infection in each country based on the analysis of 1 Million worldwide simulations of the pandemic evolution with the GLEaM model. The best estimate of the reproductive number refers to the reference value that has to be rescaled by the seasonality scaling function. The minimum scaling factor α_min_ determines the strength of the seasonality effect on the disease transmission. Here we consider α_min_ in the range [0.6–0.7], that is the best estimate obtained in Ref. [18] from the correlation analysis on the chronology of 93 countries seeded before June 18. The calibration of the model also takes into account the effects obtained by the control sanitary measures adopted in Mexico during the early stage of the epidemic [18], [30]. A thorough sensitivity analysis of the model calibration with respect to the disease natural history, initial conditions and other uncertainties in the data is reported in Ref. [18].

### Travel-related interventions and simulated scenarios

During the early stage of the outbreak, several countries implemented a variety of travel-related interventions (see Text S1 for country-specific measures and implementation details). Such measures, in addition to a spontaneous reaction of individuals to the health emergency, led to a reduction in the international traffic to/from Mexico of about 40% observed during the month of May, followed by smaller reductions in the following months, and resulting in a slow return to normality in about 3 months [31] (see Text S1). Here we consider as a *reference scenario* the one produced by the best estimates able to reproduce the initial chronology of newly infected countries (i.e. the *baseline scenario*), where in addition we take into account the empirically observed drop in air traffic, following the data reported in Table 2 of Text S1. The reference scenario is then compared to a set of hypothetical scenarios in which increasingly larger restrictions in individual mobility are considered, as well as different starting dates for the implementation of such restrictions. In addition, we also test scenarios in which country-specific air travel bans are applied, and scenarios in which ground mobility along the border between Mexico and the US is restricted (see Text S1).

It is important to stress that, contrary to previous approaches based on samples of airline mobility data [4], [9], GLEaM simulations take into account the full air travel database and the role of intra-country mobility as well as border commuting flows (e.g. across the US-Mexico border [18]). GLEaM allows for the detailed simulation of the time evolution of the spreading pattern by reproducing the infection dynamics and computing the number of travelers in each compartment. It is therefore possible to track the movement of H1N1 cases and analyze the statistics associated with arrival times, case importation, and local transmission based on many realizations that incorporate the relevant stochastic effects. The efficacy of travel-related measures is therefore measured on the timing of seeding events and resulting delays.

## Results and Discussion

### Reference scenario

**Figure 2:**
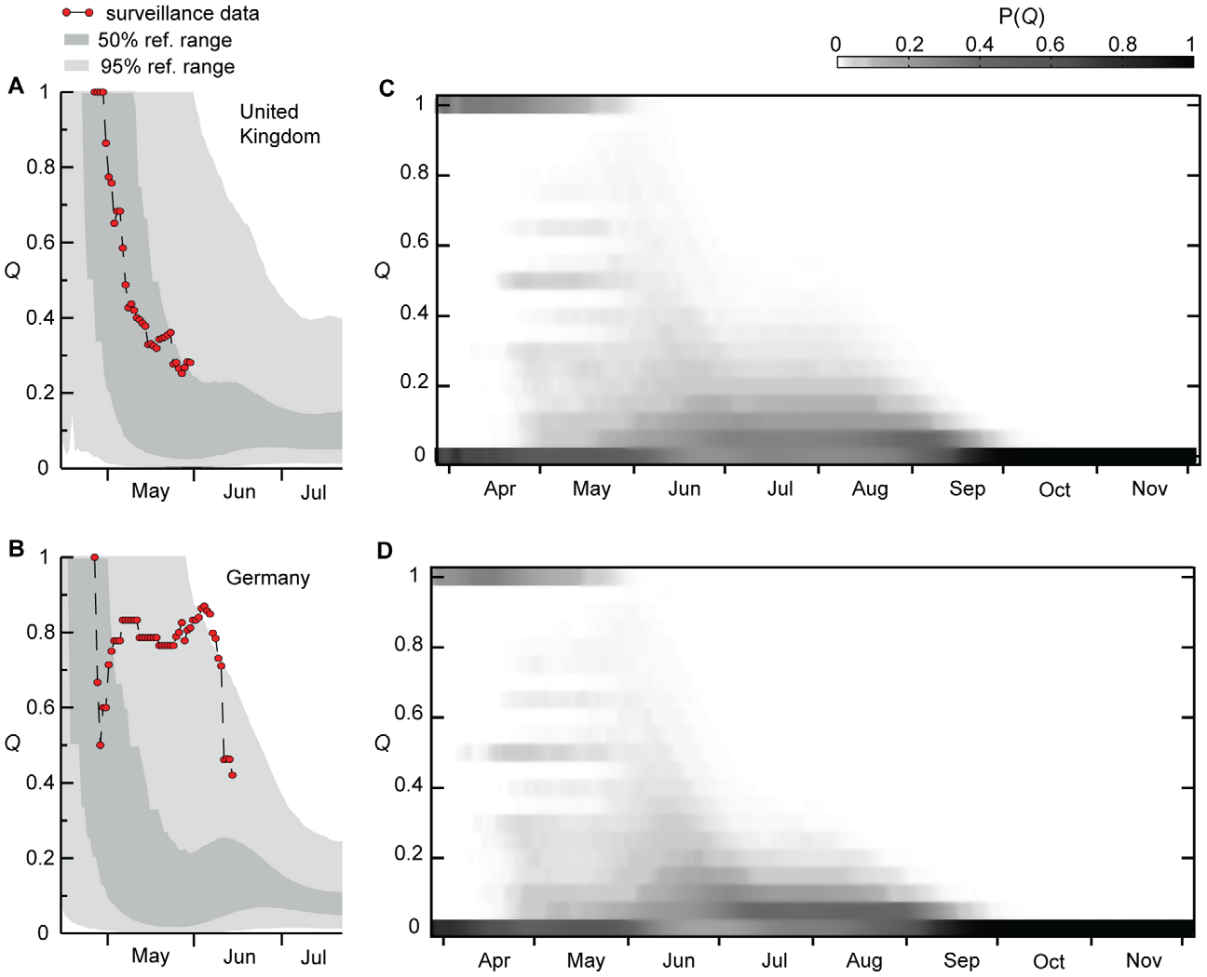
Importation of cases. **A**,**B**, Simulation results of the fraction *Q* of imported cases in United Kingdom (**A**) and Germany (**B**). The quantity *Q* is a measure of the relative weight of case importation with respect to local transmission events. The gray shaded areas show the 95% and 50% reference ranges of the simulation results obtained from 2,000 stochastic realizations. The surveillance data are indicated by red dots. **C**,**D**, Time evolution from April to November 2009 in the United Kingdom (**C**) and Germany (**D**) of the probability distribution to observe in any given realization of the epidemic the ratio *Q* between imported cases and the total number of cases. The probability distribution is obtained from the simulation of 2,000 stochastic realizations. Large values for the quantity *Q* are observed with high probability only in the early phase of the respective country's epidemic. The observed non-zero probability for a fraction of imported cases equal to zero at the early stage is due to the fact that the epidemic is imported in some cases by non-detectable individuals, such as latent and asymptomatic infectious individuals.

Figure 2 summarizes the simulation's accurate reproduction of the observed relative magnitude of imported cases in the local epidemics of newly-affected countries that validate the model. Panels A, B show cases in the United Kingdom and Germany, respectively, during the early phase of the outbreak when case-based surveillance was deployed in order to detect imported H1N1 cases and monitor local H1N1 transmission [32], [33]. Computer simulations also allow us to explore the level of stochasticity associated with the importation of infectious individuals. We keep track for each time step of each realization of the contribution of imported cases to the total prevalence in the country defined as the ratio *Q* of imported cases versus the total number of infectious individuals in the country. Since at the early stage of the epidemic there are usually large fluctuations in the number of imported local transmission cases, we measure the probability in time of observing a given ratio *Q* by averaging over 2,000 realization of the global simulation. Panels 2C, 2D show the time behavior of the probability distribution *P(Q)* clearly illustrating that the importation of cases dominates the initial phase of the epidemic in each country, which is soon followed by a sustained local transmission. The contribution of imported cases is observed at 100% with a finite probability only during the months of April-May, after which the probability distribution progressively shrinks around small values of *Q*, showing how the local H1N1 transmission starts to dominate the epidemic.

### Travel restrictions and the H1N1pdm spatial spread

The good agreement of the model with the actual data from the H1N1pdm allows us to assess the effect of the observed decline in travel flows to/from Mexico by comparing the results obtained in the reference scenario with a version of the model in which no travel reduction is considered. Compartmentalization permits tracking of the arrival of detectable (i.e. symptomatic) and non-detectable (i.e. latent or asymptomatic) infected individuals in a given country. By defining the arrival time as the date the first symptomatic case arrives in the country under study, it is possible to quantify the delay in the spreading of the epidemic. It is quite impressive to notice that the 40% drop in travel flows observed in reality only led to an average delay in the arrival of the infection in other countries (i.e. the first imported case) of less than 3 days (see Text S1 for more details). We then test whether an additional decrease in travel flows of magnitudes larger than the observed 40% would have provided an additional benefit in slowing down the propagation of the H1N1 virus across the world. We consider drops in the air travel flows connecting Mexico with the rest of the world starting on April 25 following the international alert, optimistically assuming a prompt implementation by authorities with no further delays. We also assume that the reduction is kept constant across time, differently from the empirically observed decline that successively decreased to become negligible in about 3 months.

**Figure 3:**
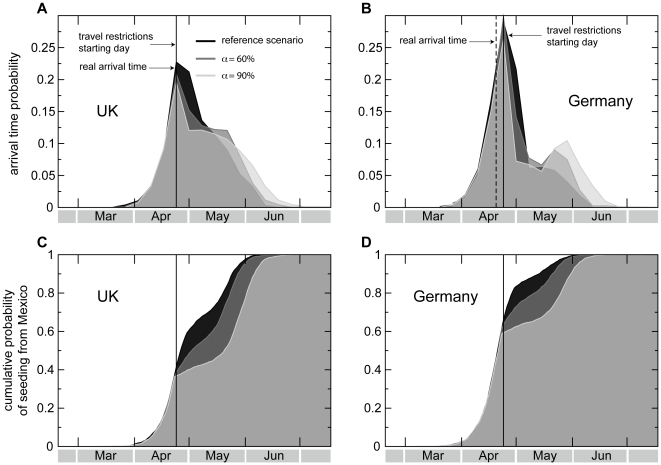
Effects of restrictions in the air travel to/from Mexico on the probability distributions of the seeding events. Travel measures imposing a reduction of 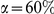 and 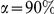 are compared to the reference scenario where the observed drop in air travel to/from Mexico is taken into account. **A**,**B**, Probability distributions of the arrival time (defined as the date of arrival of the first symptomatic case) in the United Kingdom (**A**) and Germany (**B**) for different values of 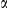. Here we consider the importation from any possible source country, not only Mexico. The vertical dotted line indicates the observed arrival time in the country, as obtained from official reports, and the vertical solid line indicates the starting date of the travel restrictions, April 25, 2009, the day after the international alert. The probability distributions are obtained from 2,000 stochastic realizations and data are binned over 7 days. Even when imposing 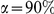, the peak of the probability distribution is not delayed with respect to the real scenario. **C**,**D**, Cumulative probability distributions of the first seeding event from Mexico to the United Kingdom (**C**) and Germany (**D**) for different values of 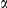. Here we consider any source of infection in the seeding event, including symptomatic cases and non-detectable infected cases, such as latent and asymptomatic, as allowed by the computational approach. The distributions are computed over 2,000 stochastic realizations. The effect of travel restrictions is very limited in delaying the time at which the cumulative distribution reaches the unit.

Figure 3 shows changes induced by travel restrictions on the simulated chronology with respect to the reference case by tracking the arrival time probability distribution. Results are reported in panels A, B of Figure 3, where application of the interventions is shown to reduce the probability values right after the peak of the distribution, with almost no change in the date of the peak. If we focus on the first arrival from Mexico, considering all possible seeding events (i.e. latent, asymptomatic, and symptomatic), we observe similar reductions in the rate of increase in the cumulative probability distribution of the seeding event, pointing to a slower rate of importation (see Figure 3C, D). However, the resulting change is not able to halt the spread.

**Figure 4:**
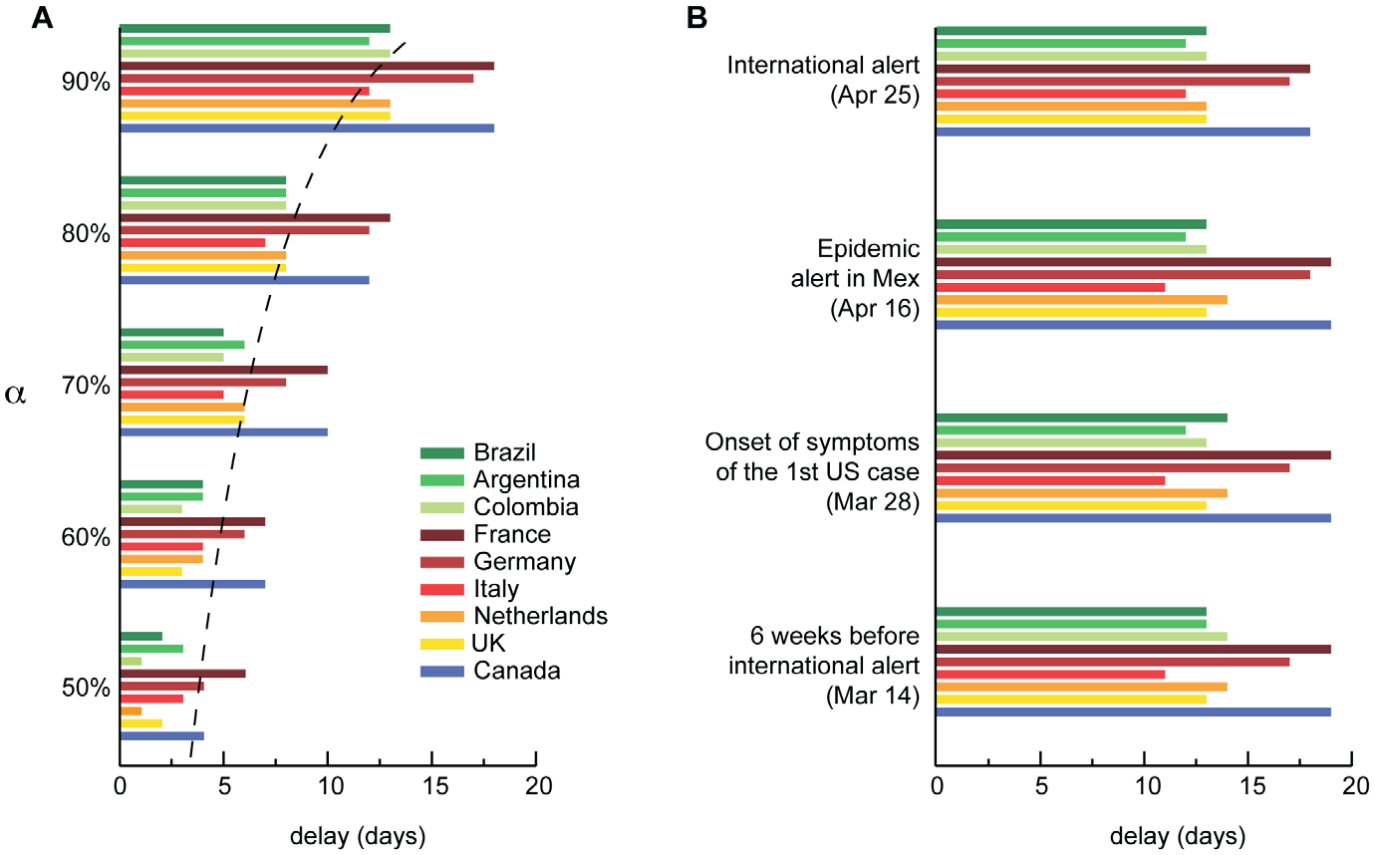
Delaying effects in the international spread. **A**, Delay in the case importation from Mexico to a given country compared with the reference scenario as a function of the travel reduction 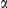. The delay is measured in terms of the date at which the cumulative distribution of the seeding from Mexico (see Figure 2) reaches 90%. The dotted line shows the logarithmic behavior relating the delay as a function of the imposed restrictions. The largest delay, gained when imposing 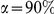, is less than 20 days for all countries. The model also considers the implementation of sanitary interventions in Mexico during the early stage that was able to damp the exponential increase of cases in the outbreak zone. Travel restrictions would therefore lead to a larger impact during this phase due to the mitigating effect on the local epidemic. If a country is seeded during this phase, the resulting delay induced by the travel restrictions would be larger, thus creating the observed differences in the resulting delays by country. **B**, as in **A**, where earlier dates for the start of the intervention are considered, has a fixed 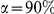: April 25, corresponding to the day after the international alert; April 16, corresponding to the epidemic alert in Mexico; March 28, corresponding to the onset of symptoms of the first case in the US; and 6 weeks before the international alert. In all these scenarios and for different countries, the delay is always less than 20 days, highlighting that even the enforcement of strong travel reduction as early as possible would have had little effect.

By considering the time at which the cumulative probability for the seeding from Mexico has reached 90%, we can calculate the delay induced by larger reductions in air travel. Figure 4A shows the delays obtained for a selection of countries. Even given the unlikely assumption of a 90% travel reduction, the resulting delay would be on the order of 2 weeks, confirming results from previous studies [4], [5], [8], [9]. This time could be used to finalize the response by the public health infrastructure of unaffected countries following the international alert, thus gaining time to enhance surveillance systems and allocate resources. Unfortunately, this timescale is insufficient to develop and distribute a vaccine. Anticipation of travel reductions following local epidemiological alerts in Mexico or the onset of symptoms from the first case in the US would lead to similar results (see panel B of Figure 4).

The exponential increase of cases in the outbreak region explains the negligible impact of travel restrictions over the course of the pandemic. Given two coupled populations with deterministic infection dynamics, the delay 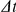
Δ*t* is a logarithmic function of the applied travel reduction of magnitude 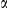, 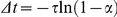, where 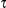 is the timescale of the epidemic's exponential growth in the seed population [34], [35]. The exponential increase of cases in the outbreak region is therefore responsible for the relatively limited delay induced by strong and lasting travel reductions. When 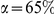, 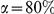 or 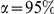 the corresponding delays become approximately 1, 1.6, and 3 times, respectively, the timescale 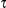 that is typically on the order of a few days. The logarithmic relation also explains more realistic situations in which the epidemic origin is characterized by spatial heterogeneity and intra-region mobility that is not subject to travel restrictions (see Text S1 for the complete analytic treatment in this case). This is the case of the H1N1 pandemic, which initially diffused within Mexico before reaching international hubs and propagating internationally.

### Global invasion threshold

**Figure 5:**
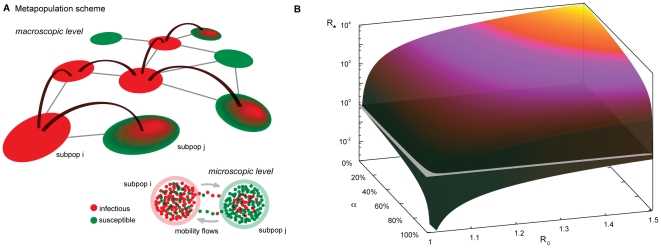
Network heterogeneity and failure of travel restrictions aimed at containment. **A**, Schematic illustration of the simplified modeling framework based on a metapopulation scheme. At the macroscopic level the system is composed of a heterogeneous network of subpopulations. At the microscopic level, each subpopulation contains a population of individuals. The infection dynamics are described by a simple compartmentalization (compartments are indicated by different colored dots in the picture). Within each subpopulation, individuals are mixed homogeneously and can migrate from one subpopulation to another following the mobility connections of the network. In this way the disease can spread at the subpopulations level. **B**, Plot of the global invasion threshold R_*_ described by Eq. (2). Here, R_*_ is plotted as a function of the basic reproductive number *R_0_* and the traffic reduction 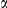, which is the parameter representing the percentage of variation in the total traffic 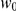 in Eq. (2). Only in the case of extremely low values of *R_0_* or extremely large values of 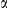 is it possible to reduce R_*_ below the threshold.

Another important question concerns the degree to which mobility restrictions are able to achieve containment at the source of the pandemic, especially in combination with timely mitigation policies in the country of origin. To this end we consider a simplified modeling framework based on a metapopulation scheme describing a network of subpopulations (nodes) coupled with mobility processes (links, see Figure 5A) whose features reproduce the topological and mobility properties of real-world transportation systems [36], [37]. We assume: (i) the large-scale heterogeneity found in the airline transportation network where the number of connections *k* departing from each airport (i.e. the degree of the node) follows a power-law distribution 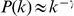; and (ii) that the observed correlations between topology and traffic, relating the number of passengers *w_ij_* traveling from airport *i* to airport *j* to the degrees *k_i_* and *k_j_* of the two subpopulations is expressed by 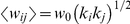, with 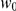 representing the mobility scale of the system [38].

Disregarding the high-resolution details of numerical approaches, this synthetic metapopulation model can now be analyzed, defining a new theoretical framework that allows for the study of epidemic containment. Starting from a single subpopulation infected at time 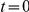, it is possible to describe the invasion dynamics at the subpopulation level in a Levins-type approach by considering the microscopic dynamics of infection and of individual travel [37]. The system is characterized by a subpopulation reproductive number R_*_. Analogous to the reproductive number *R*_0_ at the individual level, R_*_ indicates a threshold behavior of the system: if R_*_>1 the epidemic reaches global invasion; otherwise, it is contained at its source. It is possible to derive an expression for the global invasion threshold in a branching process approximation [39], [40]. Under the assumption that subpopulations having the same number *k* of connections are equivalent (i.e. the degree-block approximation, see Text S1), we define 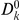 as the number of diseased subpopulations of degree *k* at generation 0 (i.e. at the beginning of the branching process). During the entire duration of the outbreak experienced by the 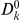 subpopulations, each of them can in principle seed some of the neighboring subpopulations thus leading to a number 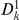 of diseased subpopulations of degree *k* at generation 1, for various values of the degree *k*. By iterating the seeding events, it is possible to describe the evolution of the number 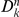 of diseased subpopulations with degree *k* at generation *n*, yielding:
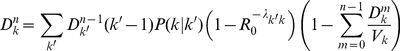
(1)


The r.h.s. of Eq. (1) describes the contribution of the subpopulations of degree *k*' at generation *n*-1 to the infection of subpopulations with degree *k* at generation *n*. Each of the 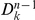 has (*k*'−1) possible connections along which the infection can proceed (−1 takes into account the link through which each of those subpopulations received the infection). In order to infect a subpopulation of degree *k*, three conditions need to occur: (i) the connections departing from nodes with degree *k*' point to subpopulations of degree *k*, as indicated by the conditional probability 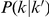; (ii) the reached subpopulations are not yet infected, as indicated by the probability 
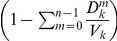
, where 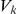 is the total number of subpopulations with degree *k*; (iii) the outbreak seeded by 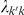 infectious individuals traveling from *k*' to *k* takes place, and the probability for this event to happen is given by 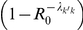 [41]. The latter term is the one that relates the microscopic dynamics of the local infection occurring within a subpopulation to the coarse-grained view that describes the disease invasion at the metapopulation level. It depends on the details of the diffusion process of individuals as well as the individual travel behavior and its interplay with the disease stages. The expression of 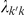 for the compartmental model here considered is derived in Text S1; by plugging it in to Eq. (1) we can derive the expression for the global invasion threshold (more details are reported in Text S1):
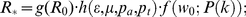
(2)where 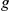
is a function that depends on the reproductive number only; 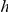
is a combination of the infection parameters 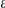, 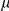, *p_a_* and *p_t_*; 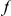
is a function of the mobility scale 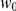 and of various moments of the distribution of the number of connections 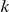
of each airport. Eq. (2) shows that R_*_ thus depends on the disease parameters, as well as the topology and fluxes of individuals' mobility.

The effect of interventions like travel restrictions, mitigation, etc., are unfortunately damped by the large topological fluctuations of human mobility patterns. The function 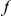 is expressed by 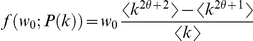 (Text S1), so that the topological heterogeneities encoded in 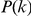 lead to very large values of the ratio 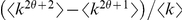, which suppresses reduction in the travel flows in 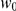, leading to values of R_*_ well above the threshold at 1 as shown by the 3D plot reported in Figure 5B. Similar conclusions apply for entry screening at the airports modeled by a reduction in the traveling probability *p_t_*, and the modeling of effective containment policies, reducing *R_0_* and the total number of cases. The large heterogeneity of human mobility patterns is therefore responsible for why travel restrictions are largely ineffective for containing an emerging pandemic.

Our analysis of the 2009 H1N1 pandemic shows that the observed decline in air travel to/from Mexico was of too small a magnitude to impact the international spread. Stricter regimes of travel reduction would have led to delays on the order of two weeks even in the optimistic case of early intervention. It is unlikely that given the ever-increasing mobility of people travel restrictions could be used effectively in a future pandemic event.

## Supporting Information

Text S1 (PDF)
