# Supplementary material for: Human Mobility Networks, Travel Restrictions, and the Global Spread of 2009 H1N1 Pandemic
Source: PLoS One. 2011 Jan 31;6(1):e16591. doi: 10.1371/journal.pone.0016591 (PMC3031602; doi:10.1371/journal.pone.0016591)
Supplement: Text S1 — (PDF) [file pone.0016591.s001.pdf]

Supporting Information Text S1
Human mobility networks, travel restrictions and the global spread of epidemics

P. Bajardi[1,2], C. Poletto[1], J. J. Ramasco[3], M. Tizzoni[1,4], V. Colizza[5,6,7], A. Vespignani[8,9,10]

1 Travel related measures and air travel flows reduction

Since the beginning of May 2009, many governments implemented different measures to prevent the A(H1N1) virus from crossing the international borders by air travel. Table 1 presents an overview of the measures adopted to slow down the spread of the A(H1N1) pandemic by controlling or limiting the international air travel flows. The measures applied were recorded by the International Airport Transport Association site [1] and were also reported by news media sources [2, 3, 4]. We list the measures from those with the highest impact on the air travel flows, aiming at isolating one country from potential sources of infection, to the less impacting measures, though capable of significantly affecting the travel behavior of passengers worldwide.

Since the end of April until the end of May 2009, four countries enforced a ban of all direct flights from Mexico: Argentina, China, Cuba and Peru. The traffic originating from these countries accounts only for 3% of the total air international traffic departing from Mexico (IATA database). The travel ban enforced by the Chinese Government affected the only direct connection with Mexico as of May 2009. The connection was not yet restored at the end of 2009 [5].

Many Asian countries enforced quarantine measures for passengers traveling from affected areas and showing influenza-like symptoms. Others, mostly in the Asian region although not exclusively, installed thermal scanners at airports' terminals prolonging their use, in some cases, until January 2010 [6]. Furthermore, immediately after the international alert, over 14 entities including governments and international organizations issued health travel warnings advising people against non-essential travel to Mexico. Even if the warnings do not physically hamper

[1]Computational Epidemiology Laboratory, Institute for Scientific Interchange (ISI), Torino, Italy
[2]Centre de Physique Théorique (CNRS UMR 6207), Marseille, France
[3]Istituto de Fisica Interdisciplinar y Sistemas Complejos IFISC (CSIC-UIB), Palma de Mallorca, Spain
[4]Scuola di Dottorato, Politecnico di Torino, Torino, Italy
[5]INSERM, U707, Paris, France
[6]UPMC Université Paris 06, Faculté de Médecine Pierre et Marie Curie, UMR S 707, Paris, France
[7]Complex Systems Lagrange Laboratory, Institute for Scientific Interchange (ISI), Torino, Italy
[8]Center for Complex Networks and Systems Research (CNetS), School of Informatics and Computing, Indiana University, Bloomington, IN, USA
[9]Pervasive Technology Institute, Indiana University, Bloomington, IN, USA
[10]Institute for Scientific Interchange (ISI), Torino, Italy

travelers' mobility, they increase people's awareness of the risks of traveling in the outbreak region, therefore contributing to further decrease the air traffic flows. Table 1 does not include other measures, such as reporting passengers with symptoms on board, checking for signs of infection through visual assessment at terminal checkpoints and requiring passengers to fill a health status declaration form, which were widely adopted by many countries.

All these measures, with the addition of self-imposed limitations due to the pandemic concern, led to the reduction of the number of passengers flying to and from Mexico. Table 2 reports the monthly number of passengers (in millions) who traveled to and from Mexico on international flights from April 2009 to December 2009. Data are obtained from the site of the *Secretaría de Comunicaciones y Transportes* (Mexican Department of Transportation) [7]. If compared to 2008, the drop in the number of passengers has been particularly high during May 2009 following the A(H1N1) pandemic alert issued by the WHO. The international air traffic to/from Mexico returned to normality in about 3-4 months.

Adopted measure	Country	Time period
**Mexican flight ban**	Argentina	from April 28 to May 14, 2009 [9, 10]
	China	from May 2, 2009 [5, 11]
	Cuba	from April 30 to May 31, 2009 [12]
	Peru	from April 29 to May 13, 2009 [13, 14]
**Quarantine of passengers**	China, Hong Kong Japan, Taiwan, Singapore.	discontinued by all countries by July 2009.
**Thermal screening**	Bulgaria, Chile, China, Ecuador, Hong Kong, India, Jordan, Lebanon, Malaysia, Qatar, Singapore, Thailand, UAE.	discontinued by all countries by January 2010.
**Health travel warnings**	Bosnia, Bulgaria, Canada, Chile, Colombia, France, Germany, Korea, Russia, Turkey, United States, UK Venezuela, Vietnam.	discontinued by all countries by June 2009.

Table 1: Known measures adopted worldwide against the pandemic spread [1, 2, 3, 4].

Month		Passengers on international flights, Mexican airline (mil.)	Passengers on international flights, foreign airline (mil.)	Total
April	2008	0.6	1.7	2.3
	2009	0.6	1.6	2.2
	var.	=	−6%	−4%
May	2008	0.6	1.5	2.1
	2009	0.4	0.7	1.1
	var.	−33%	−53%	−48%
June	2008	0.6	1.5	2.1
	2009	0.5	1.1	1.6
	var.	−17%	−27%	−24%
July	2008	0.8	1.6	2.4
	2009	0.7	1.3	2.0
	var.	−12%	−18%	−17%
August	2008	0.7	1.5	2.2
	2009	0.7	1.2	1.9
	var.	=	−20%	−14%
September	2008	0.5	0.9	1.4
	2009	0.5	0.9	1.4
	var.	=	=	=
October	2008	0.6	1.1	1.7
	2009	0.5	1.0	1.5
	var.	−17%	−9%	−12%
November	2008	0.6	1.4	2.0
	2009	0.6	1.3	1.9
	var.	=	−7%	−5%
December	2008	0.7	1.6	2.3
	2009	0.7	1.5	2.2
	var.	=	−6%	−4%

Table 2: Mexican air traffic statistics April - December 2009 [7]. In the reference scenario the traffic reduction has been modeled according to these values. They were adjusted to take into account the economic recession that has produced an overall drop in the traffic of 2009 with respect to 2008. The average drop was estimated to be equal to 6% based on the traffic values of the months prior to the pandemic.

2 The GLobal Epidemic and Mobility model

GLEaM [15, 16] is a global epidemic and mobility structured metapopulation model. It is based on a meta-population approach [17, 18, 19, 20, 21, 22, 23, 24, 25, 26, 27, 28] in which the world is divided into geographical regions defining a subpopulation network where connections among subpopulations represent the individual fluxes due to the transportation and mobility infrastructure. GLEaM integrates three different data layers [15, 16]: (i) the population layer that describes the demographic composition of the model, (ii) the transportation mobility layer, and (iii) the epidemic layer which models the infection transmission within each subpopulation. Both the traveling and the infection spreading are discrete-time stochastic processes with a time resolution of one day, and the individuals are regarded as integer random variables. By varying the details and the parameters of the model, it is possible to simulate the global spreading of many different kinds of infectious diseases. In the following, we will describe first the general architecture of the model (i.e. the three layers), and then the aspects of the specific application of the model to the A(H1N1) influenza pandemic.

2.1 Demographic, mobility and epidemic layers

Population layer
The demographic layer is based on the high-resolution population database of the "Gridded Population of the World" project of SEDAC (Columbia University) [29]. We extracted from this database the world population with a geographical resolution of 15×15 minutes of arc. The census areas associated to the subpopulations of the metapopulation system are obtained by a Voronoi-like tessellation of the Earth surface taking as centers the coordinates of 3362 airports [15, 16].

Mobility layer
The transportation mobility layer integrates air travel mobility from the International Air Transport Association (IATA) [30] and OAG [31] databases, and the commuting patterns worldwide obtained from the data collected and analyzed from the census of more than 30 countries [15, 16]. The number of passengers traveling each time step is an integer random variable defined by the actual data from the airline transportation database. Short range commuting between subpopulations is modeled with a time scale separation approach that defines the effective force of infections in connected subpopulations [15, 16, 32, 33].

Epidemic layer
The infection dynamics within each subpopulation is modeled as a homogeneous mixing process with a compartmental approach specific for the disease considered. For the case of the A(H1N1) pandemic, we select the $\{S, E, I_a, I_{st}, I_{snt}, R\}$ compartmentalization [34, 35] described in the main paper (see Fig. 1 of the main paper for a schematic representation of the compartmental model). The parameters that regulate the transitions between the compartments along with their descriptions and values are listed in Table 3. The generation time G_t (the sum of the latency and infectious period, ϵ^{-1} and μ^{-1}) and the basic reproductive number are set according to the estimates of [16] (see the following Subsection). All the other parameters are assumptions of the model based on estimates found in the literature [35, 36, 37, 38]; an extensive sensitivity analysis was performed

in Ref. [16] in order to assess the effects that variations on these values can have on the simulation results and the explored ranges of each parameter are reported in Table 3.

Parameter	Description	Best Estimate	Interval Estimate
R_0	basic reproductive number	1.75	$1.64 - 1.88$
G_t	mean generation time (days)	3.6	$2.2 - 5.1$
α_{min}	minimum seasonality rescaling	0.65	$0.6 - 0.7$
		Assumed values	Sensitivity analysis range
α_{max}	maximum seasonality rescaling	1.1	$1.0 - 1.1$
r_β	relative infectiousness of asymptomatic individuals	0.5	$0.2 - 0.8$
p_t	probability of becoming a traveling symptomatic individual	0.5	$0.4 - 0.6$
p_a	probability of becoming an asymptomatic individual	0.33	$0.33 - 0.5$

Table 3: Epidemic parameters along with their description and value. For R_0 we report the 95% reference range. G_t interval is defined by the range of plausible constrained values sampled in the Monte Carlo approach that satisfy a likelihood ratio test at the 5% level. The α_{min} interval is the best-fit range within the minimal resolution allowed by the Monte Carlo sampling.

2.2 2009 H1N1 calibration and parameters' estimate

In order to simulate the H1N1 pandemic, the model was calibrated on the epidemiological data available at the early stage of the pandemic evolution. The procedure used to estimate the parameters and the model details specific for the H1N1 case are described in Ref. [16] and will be summarized in this section.

Seasonality is considered in the model by means of a sinusoidal forcing of the reproductive number, with a scaling factor ranging from α_{min} during Summer season to α_{max} during Winter season [21]. No rescaling is applied in the tropical regions. We assumed a value for $\alpha_{max} = 1.1$ (exploring in the sensitivity analysis values between 1.0 and 1.1) and we estimated from empirical data the value of α_{min}. Initial conditions were defined by setting the start of the epidemic near La Gloria, Mexico, on February 18 2009 as in Ref. [16], analogously to other works [36] and also following available data from official sources [39]. Finally, in order to reproduce the timeline of events characterizing the early stage of the epidemic unfolding, we included in our model the intervention measures implemented by the Mexican authorities in the attempt to control the outbreak in Mexico. The measures implemented aimed at increasing social distance; they were

simulated by considering a lower basic reproduction ratio in Mexico during this period of time [16, 40].

The seasonal transmission potential of the new H1N1 influenza was estimated in Ref. [16] from epidemiological data collected at the early stage of the pandemic evolution. The estimation procedure was based on the detailed knowledge of human mobility patterns. In Ref. [16] we used GLEaM to simulate the worldwide evolution of the pandemic and performed a maximum likelihood analysis of the parameters against the actual chronology of newly infected countries. The method shifts the burden of estimating the disease transmissibility from the incidence data, suffering notification/surveillance biases and dependent on country specific surveillance systems, to the more accurate data of the early case detection in newly affected countries. The seasonal transmission potential of the H1N1 strain was assessed in a two-step process that first estimates R_0, the reproductive number in the Tropics region, where seasonality is assumed not to occur, by focusing on the early international seeding by Mexico, and then estimates the degree of seasonal dumping factor α_{min}, by examining a longer time period of international spread to allow for seasonal changes.

The estimation of the reproductive number is performed through a maximum likelihood analysis of the model fitting the data on the arrival date of the first symptomatic in 12 countries directly seeded from Mexico. Given a set of values of the disease parameters, we produced 2×10^3 stochastic realizations of the pandemic evolution worldwide for each R_0 value. Our model allows the tracking of the importation of each symptomatic individual as observables of the simulations. This allows us to obtain numerically with a Monte Carlo procedure the probability distribution $P_i(t_i)$ of the importation of the first infected individual or the first occurrence of the onset of symptoms in each country i at time t_i. This allows us to define a likelihood function $\mathcal{L} = \prod_i P_i(t_i^*)$, where t_i^* is the empirical arrival time from the H1N1 chronological history in each of the selected countries. Given that the countries are directly seeded from Mexico the variables t_i are conditional independent and thus we can factorize $\mathcal{P}(\{t_i\}) = \prod_i P_i(t_i^*)$. The transmission potential is estimated as the value of R_0 that maximizes the likelihood function $\mathcal{L}$, for a given set of values of the disease parameters.

In order to quantify the degree of seasonality observed in the current epidemic, we estimated the minimum seasonality scaling factor α_{min} of the sinusoidal forcing by extending the chronology under study and analyzing the whole data set composed of the arrival dates of the first infected case in the 93 countries affected by the outbreak as of June 18. We studied the correlation between the simulated arrival time by country and its corresponding empirical value, by measuring the regression coefficient between the two datasets. Given the extended time frame under observation, the arrival times considered in this case are expected to provide a signature of the presence of seasonality. They included the seeding of new countries from outbreaks taking place in regions where seasonal effects might occur, as for example in the US or in the UK. The regression coefficient was found to be sensitive to variations in the seasonality scaling factor, allowing discrimination of the α_{min} value that best fits the real epidemic. A detailed presentation of this analysis is reported in [16].

Along with the parameter calibration, a systematic sensitivity analysis was performed on the assumptions used in the model [16], including the effect of the control measures in Mexico and the initial date of the epidemic. Variations on the values of p_a, p_t, r_β and α_{max} were also tested. Finally, we explored a shift of 7 days earlier for all arrival times available from official reports in

order to analyze the effect of a possible late/missed detection of symptomatic individuals. The R_0 estimate resulted to be quite robust to the other model assumptions.

Table 3 reports the reference values of the epidemiological parameters including the best estimates and the range explored with the sensitivity analysis for the assumed values.

2.3 Simulations of the 2009 H1N1 pandemic influenza

The model calibrated as described in the previous section was used in the analysis presented in this paper. As a further ingredient, here we account for the registered drop in the international traffic to/from Mexico in the four months from May to August. The data reported in Section 1 are used in order to estimate this latter effect month by month. We have adjusted the values of the Table 2 to take into account the economic recession that has produced an overall drop in the traffic of 2009 with respect to 2008. The average drop was estimated to be equal to 6% based on the traffic values of the months prior to the pandemic, and was then subtracted to the numbers reported in the Table.

All these parameters and model details represent our reference scenario for the A(H1N1) pandemic evolution. This is compared to a range of additional scenarios where we test travel related interventions of various kind. In particular we explored: reductions of different magnitude of the traffic to/from Mexico including no reduction at all; different starting dates of the traffic reduction; air traffic ban in Argentina concerning flight to/from Mexico; border closure between Mexico and US. The results of the comparison between these scenarios and the reference are described in detail in the main paper and in the following sections.

3 Impact of the travel related measures

GLEaM allows us to assess the impact of the travel related measures that were implemented during the outbreak of the 2009 H1N1 pandemic. We analyzed the effects of the international travel ban imposed in Argentina and of the overall drop observed from May to September in the travel flows to/from Mexico. Besides the observed drops in the airline traffic, we also explored the impact of the closure of US-Mexico border, a travel related intervention measure that was highly debated after the pandemic alert diffusion [41, 42, 43].

In order to address the impact of the overall reduction in the international passenger flows to/from Mexico registered after the pandemic alert, we compared two scenarios. Our reference scenario where the actual drop is considered, and the baseline scenario where the traffic is kept invariant after the pandemic. Fig. 1a and Fig. 1b show the comparison of the seeding time distributions (i.e., the arrival of the first exposed or infectious individual from Mexico) obtained for the two scenarios, considering, as in the main paper, United Kingdom and Germany as paradigmatic examples. These results highlight the almost negligible impact of this drop despite its large magnitude.

The same analysis was performed to investigate the benefit produced by the travel ban imposed by Argentina in delaying the epidemic spreading in the country. We simulated the application of the ban and we compared this scenario with the reference scenario. In Fig. 1c, the

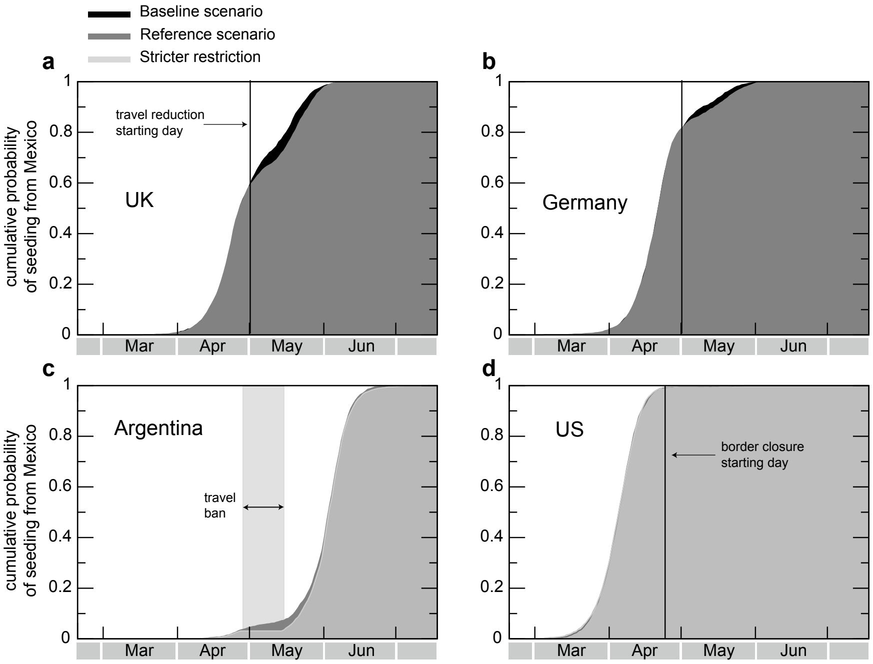

Figure 1: Plots of the seeding time cumulative distribution under different intervention scenarios. The seeding time is defined as the date of arrival of the first exposed or symptomatic case from Mexico. Panels a and b: comparison of the reference scenario with a scenario in which the air passenger flows is kept constant during the epidemic unfolding. The two countries UK (a) and Germany (b) are taken as examples. The starting time for the traveler flow reductions is highlighted in the plots by a vertical line. Panel c: comparison of the reference scenario with a scenario in which direct air connections between Argentina and Mexico have been suspended for the time period indicated by the gray area. Panel d: Effect of the border closure between Mexico and the US. The vertical line indicates the starting day of the intervention measure, April 24.

cumulative distributions of seeding time are shown for both scenarios. The comparison points out that such intervention measure, even if extreme, is quite ineffective.

In order to investigate the role of the intra-country commuting between Mexico and the United States, we simulated the hypothetical scenario in which a closure of the border US-Mexico was enforced, starting at the end of April, in response to the pandemic alert. This allows us to analyze the impact in the spreading of the disease within the US due to this intervention measure. In Fig. 1d the cumulative distribution of seeding times in the US is shown for the reference scenario and the scenario without US-Mexico commuting. The comparison between the two cases shows the inefficacy of such intervention measure.

4 Travel restrictions and delay in arrival times

Here we study the value of travel restrictions as control measures by estimating their efficacy in terms of the delay induced in the arrival time of the disease in each geographical location (census area, in GLEaM's notation). Our starting point is the analytical work of Scalia-Tomba and Wallinga [44] which determines the functional relationship between delay and traffic reduction for the case of two connected populations with a deterministic infection evolution within each of them. Our contribution is to extend this work to the two more realistic cases in which (i) the infection transmission within each location is modeled with a stochastic SIR process, and (ii) a complex spatial structure characterize the regions of origin and destination.

Our analytical results are confirmed by GLEaM's numerical simulations. A detailed comparison between theoretical predictions and simulations is shown at the end of this Section.

4.1 Model with two subpopulations

In the following we extend the results of Ref. [44] to the case of a stochastic SIR infection dynamics within each location. We first start with the case studied in Ref. [44]. Consider two subpopulations A and B. The travel of infectious individuals from A (the origin of the epidemic) to B can be regarded as a non-homogeneous Poisson process with the average number of infectious individuals arriving in B at each instant t being $I(t)\,\omega\,dt$, where ω is the mobility rate from A to B and $I(t)$ is the number of infectious individuals in A at time t. The cumulative distribution of arrival time of the first infectious individual at B can be written as follows [45, 46, 47]

$$P(t) = 1 - e^{-\omega \int_0^t ds\, I(s)}. \quad (1)$$

Note that if the integral $\int_0^\infty ds\, I(s)$ is finite the arrival time distribution is not strictly normalized to one. The remaining probability, $p_{\text{no outbreak}} = e^{-\omega \int_0^\infty ds\, I(s)}$, corresponds to the instances in which the disease does not arrive at B (Ref. [48] specifically address this point). Nevertheless $p_{\text{no outbreak}} \simeq 0$ whenever R_0 is high enough (as for instance in the case of the A(H1N1) influenza) and $\omega N >> 1$ where N is the population of A (condition satisfied in GLEaM for all the census areas and all the connections). The distribution given in Eq. (1) can be generalized to the case in which a reduction of α in the travel occurs at time t_0. We account for this effect by rescaling ω by a factor $1-\alpha$. For $t > t_0$, the expression for $P(t)$ becomes then

$$P_\alpha(t) = 1 - e^{-\omega \int_0^{t_0} ds\, I(s) - (1-\alpha)\,\omega \int_{t_0}^t ds\, I(s)}. \quad (2)$$

Consider now the time necessary for the location B to be seeded with a certain probability $P(t) = \zeta$. As done in Ref. [44], the difference in time to reach the same probability when travel reductions are in place can be used to evaluate their effectiveness to delay the disease spread. Such delay Δt can be obtained by solving the equation $P(t) = P_\alpha(t + \Delta t) = \zeta$, and, for the case in which $I(t) \propto e^{t/\tau}$, we have that

$$\Delta t(\alpha) = -\tau \ln(1-\alpha) + \tau \ln\left(1 - \alpha\, e^{(t-t_0)/\tau}\right), \tag{3}$$

where τ is the time scale of the exponential growth of the number of infectious individuals in A. The second term of Eq. (3) is small whenever $t - t_0 \gg \tau$. Under this condition, the logarithmic relation by Scalia-Tomba and Wallinga is recovered [44]

$$\Delta t(\alpha) = -\tau \ln(1-\alpha). \tag{4}$$

We proceed now to adapt this analysis to the case in which the infection transmission within the subpopulations is governed by an SIR stochastic dynamics. More complicated stochastic compartmental models can probably be subjected to a similar analysis even if it becomes more cumbersome. We will show that the expression for Δt of Eq. (4) is still valid for the stochastic SIR.

Since the contagion process within A is stochastic, we must consider a full distribution of infectious individuals at time t $p_t(I)$ instead of a single value $I(t)$. More precisely, since the cumulative arrival time distribution of Eq. (1) depends on the integral of $I(t)$, we will work with the joint distribution $p_t(I, \mathcal{I})$ where $\mathcal{I}(t) = \int_0^t ds\, I(s)$. In this new framework, Eq. (1) becomes

$$P(t) = \int p_t(I, \mathcal{I})\,(1 - e^{-\omega \mathcal{I}})\, d\mathcal{I}\, dI. \tag{5}$$

The explicit expression of $p_t(I, \mathcal{I})$ is therefore needed in order to perform the integral. Since our interest lies in the early dynamics of the epidemic in A, we can obtain $p_t(I, \mathcal{I})$ by approximating the contagion process with a birth-and-death process [49]. In this process, infectious individuals are introduced in the system with a constant generation rate (the transmission rate β) and disappear with a constant death rate (the recovery rate μ). This approach is analogous to the exponential growth considered in the analysis of the deterministic contagion. Note that under these assumptions the compartmental models SIS and SIR are equivalent. The possible transitions of the system of the birth-and-death process in the bivariate space of the vector $(I, \mathcal{S})$ are given by

$$\begin{array}{lll} (I,\mathcal{S}) \to (I+1, \mathcal{S}+1), & \text{with prob} & \beta I dt + O(dt), \\ (I,\mathcal{S}) \to (I-1, \mathcal{S}), & \text{with prob} & \mu I dt + O(dt), \\ (I,\mathcal{S}) \to (I, \mathcal{S}), & \text{with prob} & 1 - (\beta+\mu)\, I dt + O(dt), \end{array} \tag{6}$$

where $\mathcal{S}$ stands for the size of the epidemic, which can be related to $\mathcal{I}$ as $\mathcal{S} \approx \beta\, \mathcal{I}$. The forward Kolmogorov equation associated to the process reads [50]

$$\begin{cases} \partial_t p_t(I,\mathcal{S}) = \beta\,(I-1)\, p_t(I-1, \mathcal{S}-1) + \mu\,(I+1)\, p_t(I+1, \mathcal{S}) - (\beta+\mu)\, I\, p_t(I, \mathcal{S}) \\ \partial_t p_t(0, \mathcal{S}) = \mu\, p_t(1, \mathcal{S}), \end{cases} \tag{7}$$

with the initial conditions $p_0(I, \mathcal{S}) = \delta_{I,I_0} \delta_{\mathcal{S},0}$. This equation can be solved by introducing the generating function associated to $p_t(I, \mathcal{S})$,

$$G_t(u, w) = \sum_{I,\mathcal{S}} u^I\, w^{\mathcal{S}}\, p_t(I, \mathcal{S}). \tag{8}$$

Taking the partial derivative $\partial_t G_t(u, w)$ and substituting $\partial_t p_t(I, \mathcal{S})$ with the second term of Eq. (7), we obtain the following differential equation for $G_t(u, w)$

$$\partial_t G = [\beta\, w\, u^2 - (\beta + \mu)\, u + \mu]\, \partial_u G, \tag{9}$$

whose solution is [50]

$$G_t(u, w) = \left[\frac{\alpha_1(\alpha_2 - u) + \alpha_2(u - \alpha_1) e^{\beta(\alpha_1 - \alpha_2) w t}}{(\alpha_2 - u) + (u - \alpha_1) e^{\beta(\alpha_1 - \alpha_2) w t}} \right]^{I_0}. \tag{10}$$

Here α_1 and α_2 are the solutions of the following second order equation in u

$$\beta\, w\, u^2 - (\beta + \mu)\, u + \mu = 0. \tag{11}$$

The generating function $G_t(u, w)$ contains all the information regarding the distribution $p_t(I, \mathcal{S})$. An explicit expression of this probability distribution is not necessary since all observables can be computed directly from it.

By using the definition of $G_t(u, w)$ into Eq. (5), we have that

$$P(t) = \int \left(1 - e^{-\frac{\omega}{\beta}\mathcal{S}}\right) p_t(I, \mathcal{S})\, dI\, d\mathcal{S} = 1 - G_t\left(1, e^{-\frac{\omega}{\beta}}\right). \tag{12}$$

Therefore the explicit formula for $P(t)$ can be easily calculated. The full expression is quite complicated and here we report an approximate functional form valid in the limit $\omega << \beta$ (a condition that is satisfied in realistic situations). In this limit, $P(t)$ can be approximated as

$$P(t) \simeq 1 - \left[\frac{\mu R_0 (R_0 - 1)^2 + \omega(R_0^2 - R_0 + 1) + \omega R_0 e^{\mu(R_0 - 1)t}}{\mu R_0 (R_0 - 1)^2 + \omega + \omega R_0^2 e^{\mu(R_0 - 1)t}} \right]^{I_0}, \tag{13}$$

with $R_0 = \beta/\mu$. Despite this expression is quite different from the Gumbel distribution obtained for the deterministic epidemic dynamics [45, 46], it gives us the same logarithmic relation for the delay as a function of $1 - \alpha$, where α is the drop in the travel flow. This can be easily shown in the case of travel reductions starting at $t_0 = 0$, since $P_\alpha(t)$ is obtained from $P(t)$ replacing ω with $(1 - \alpha)\, \omega$. Solving the equation $P(t) = P_\alpha(t + \Delta t) = \zeta$, with respect to the delay Δt, we obtain that, except for terms of the order of $O(\omega)$, Eq. (4) still holds in this case.

For $t_0 > 0$, the calculations are a little more laborious but the results are similar. The expression for $P_\alpha(t)$ must be modified to include the contribution to the arrival probability of the infectious individuals in the source before and after the travel reductions occur. This means that

$$\begin{aligned} P_\alpha(t) &= \int \left(1 - e^{-\frac{\omega(1-\alpha)}{\beta}\mathcal{S}_1 - \frac{\omega\,\alpha}{\beta}\mathcal{S}_2}\right) p_{t_0}(I_1, \mathcal{S}_1)\, p_t(I_2, \mathcal{S}_2)\, dI_1\, d\mathcal{S}_1\, dI_2\, d\mathcal{S}_2 \\ &= 1 - G_{t_0}\left(1, e^{-\frac{\omega\,(1-\alpha)}{\beta}}\right) G_t\left(1, e^{-\frac{\omega\,\alpha}{\beta}}\right). \end{aligned} \tag{14}$$

Since the evolution of the system is described by a Markov process, the two contributions in the integral can be regarded as independent. Once again we obtain Δt from equating $P(t) = P_\alpha(t + \Delta t) = \zeta$ and we find the same logarithmic dependence between Δt and $1 - \alpha$, as leading term in an expansion in ω:

$$\Delta t(\alpha) = -\frac{1}{(\beta - \mu)} \ln(1 - \alpha) + \frac{1}{(\beta - \mu)} \ln\left(1 - \alpha\, e^{-(\beta - \mu)\,(t - t_0)}\right). \tag{15}$$

4.2 Model with a complex mobility network among subpopulations

Here we consider a case similar to the seed of the A(H1N1) pandemic where the origin of the outbreak itself is characterized by a spatial structure and connections among subpopulations. A traffic drop α would then act upon all the transport connections between the country of origin and that of destination, but will not affect the mobility connections within the country of origin. The complex spatial structure of the transportation network in the source country plays an important role in the timing of the disease propagation. Nevertheless, the analytic calculations, that we are going to illustrate next, show that the logarithmic relation between Δt and α of Eq. (4) is still valid in the first order of approximation.

In order to generalize our previous framework to this case, consider a country B connected to a set of cities A_k of the seeding country. The internal spatial structure of the transportation network in B is not relevant for our calculations: the internal connections indeed do not count since the arrival time of the disease in B corresponds to the first arrival from A at any of the B census areas. We define ω_k the travel rates from a census area A_k in A to the target country B. We assume that the epidemic starts in a given city of A and spreads both within and outside the country. Let t_k indicate the arrival time of the epidemic at the A_k subpopulation. As in the previous Subsection, we define $\mathcal{I}_k(t) = \int_{t_k}^{t} ds\, I_k(s)$, where $I_k(t)$ corresponds to the number of infectious individuals in A_k at time t, and t_0 as the starting date for the travel reductions. The expression for $P_\alpha(t)$ is therefore equal to

$$P_\alpha(t) = \int_{\{I_k,\mathcal{I}_k\}} \left(1 - e^{-\sum_k \omega_k [\alpha \mathcal{I}_k(t_0) + (1-\alpha)\mathcal{I}_k(t)]}\right) p_{t_0}(\{I_k,\mathcal{I}_k\})\, p_t(\{I_k,\mathcal{I}_k\}) \prod_k dI_k\, d\mathcal{I}_k, \quad (16)$$

where we have assumed that the stochastic properties of the spreading process can be described by the distributions $p_t(\{I_k,\mathcal{I}_k\})$, as we did in the previous Subsection. Notice that, since travel reductions apply only to the connections between countries A and B, the evolution of the epidemic inside A is not expected to change by the introduction of such measures and so the distributions $p_t(\{I_k,\mathcal{I}_k\})$ are invariant.

To make calculations simpler, we consider a deterministic SIR. In this case, we can write $I_k(t) = C_k\, e^{(t-t_k)/\tau}$, where C_k is a constant related to initial conditions. Therefore we have

$$\mathcal{I}_k(t) = \tau C_k \left(e^{(t-t_k)/\tau} - 1\right) \Theta(t - t_k). \quad (17)$$

This last expression differs from zero only for times $t > t_k$, as guaranteed by the Heaviside function $\Theta(x)$, that is equal to zero when the argument is negative and equal to one otherwise. The stochasticity of the spreading process now lies in the arrival times t_k and in the initial conditions C_k. This means that the distributions $p_t(\{I_k,\mathcal{I}_k\})$ can be expressed as $p_t(\{t_k, C_k\})$. Substituting it in Eq. (16) we obtain

$$P_\alpha(t) = \int_{\{t_k,C_k\}} f_\alpha(t, t_k, C_k)\, p_t(\{t_k, C_k\}) \prod_k dt_k dC_k, \quad (18)$$

where

$$f_\alpha(t, t_k, C_k) = \left(1 - e^{-\sum_k \tau \omega_k C_k [\alpha \Theta(t_0 - t_k)(e^{(t_0-t_k)/\tau} - 1) + (1-\alpha)\Theta(t - t_k)(e^{(t-t_k)/\tau} - 1)]}\right). \quad (19)$$

As before, in order to find an expression for the delay Δt we solve the equation $P(t) = P_\alpha(t + \Delta t) = \zeta$. Since

$$f_\alpha(t+\Delta t, t_k, C_k) - f_0(t, t_k, C_k) \geq 0 \tag{20}$$

this equation simply reduces to

$$f_\alpha(t+\Delta t, t_k, C_k) = f_0(t, t_k, C_k) \quad \Rightarrow$$

$$\begin{aligned} &\sum_k \tau\,\omega_k C_k\, \Theta(t-t_k)\,(e^{(t-t_k)/\tau}-1) = \\ &\sum_k \tau\,\omega_k C_k \left[\alpha\,\Theta(t_0-t_k)\,(e^{(t_0-t_k)/\tau}-1) + (1-\alpha)\,\Theta(t-t_k)\,(e^{(t-t_k)/\tau}-1)\right]. \end{aligned} \tag{21}$$

Through straightforward calculations we thus find the expression for the delay Δt induced by a travel reduction α:

$$\Delta t(\alpha) = \tau\{-\ln(1-\alpha) + \ln(1-\mathcal{E})\}, \tag{22}$$

with

$$\mathcal{E} = -\alpha \frac{\sum_{k|t>t_k} \omega_k\, C_k\, \left[1 + \Theta(t_0-t_k)\,(e^{(t_0-t_k)/\tau}-1)\right]}{\sum_{k|t>t_k} \omega_k\, C_k\, e^{(t-t_k)/\tau}}, \tag{23}$$

This term becomes negligible when t is large enough compared with t_0 and with the t_k of the subpopulations of A. In this case, we recover the expression

$$\Delta t(\alpha) = -\tau \ln(1-\alpha). \tag{24}$$

4.3 Comparison between analytical and numerical results

The logarithmic relation between the delay Δt and the traffic drop α can be also tested in GLEaM's numerical simulations. To perform the comparison between theoretical expectations and simulations we considered two possible scenarios. The full scenario described in Subsection 2.3, and one in which we do not consider the control sanitary measures in Mexco. To both scenarios, we apply travel reductions of magnitude α, starting on April 25, 2009. The simpler scenario, with no sanitary measures, is considered as a benchmark scenario where no change in the exponential growth of the epidemic is induced by additional interventions.

We consider different values of α and compute for each of them the delay in reaching the probability of seeding $\zeta = 90\%$. This procedure was carried out at a country level for several destination countries. The theoretical predictions agree well with the numerical results for $\Delta t(\alpha)$ obtained with GLEaM, as can be seen in Fig. 2 where the logarithmic relation of Eq. (4) is plotted against the simulation results. For the $SEI_tI_{nt}I_aR$ compartmental model used in GLEaM, the time scale of the exponential growth is given by the relation [15, 16, 51]

$$\tau^{-1} = \frac{1}{2}\left[\sqrt{(\epsilon+\mu)^2 + 4\epsilon(\beta(1-p_a+p_a r_\beta)-\mu)} - (\mu+\epsilon)\right], \tag{25}$$

and is not affected by seasonality. Despite the control sanitary measures in Mexico alter the epidemic exponential behavior, the logarithmic behavior of Δt with $1-\alpha$ still holds to a good approximation for the full model described in Subsection 2.3 and considered in the main paper. As

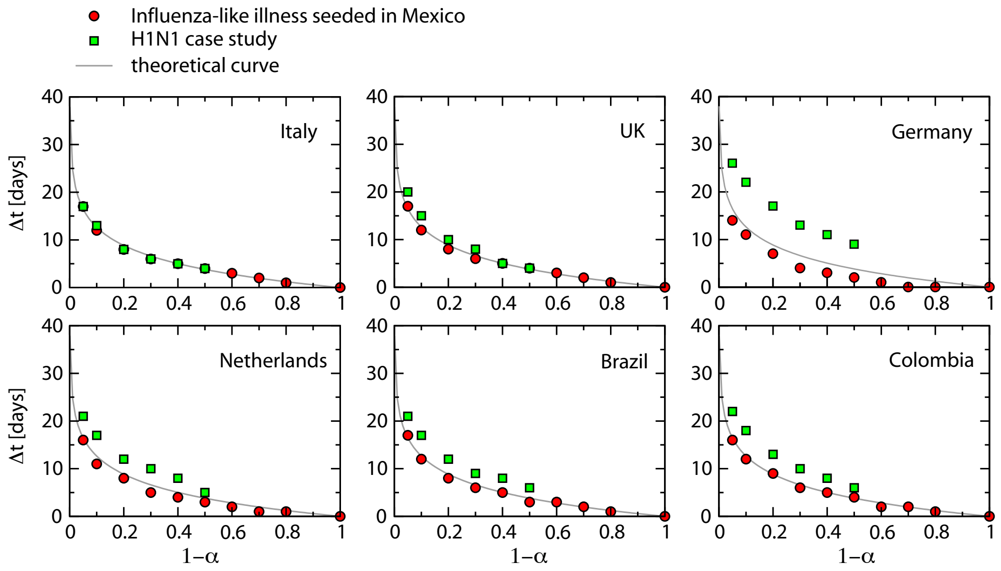

Figure 2: Delay Δt as a function of the travel reductions $1-\alpha$ to reach a seeding certainty level from Mexico of $\zeta = 90\,\%$. The results for the simplified model (without sanitary measures implemented in Mexico) are plotted as red circles, while those for the full model as green squares. The continuous curves correspond to the theoretical predictions of Eq. (4).

Fig. 2 shows, the interplay between the travel restrictions and the control measures within Mexico is quite complex. By dumping the exponential increase of cases in Mexico, the sanitary control measures enhance the delay produced by travel restrictions, as it is quite evident for the case of Germany. This effect is, however, memoryless. Indeed, the delay measured after the sanitary controls are released follows the logarithmic relation as shown by the curve of the UK.

5 Global Invasion Threshold

After a brief introduction of the theoretical framework, we complete the description given in the main paper of the analytical derivation of R_*.

We consider a synthetic metapopulation system whose demographic and mobility properties are set in order to reproduce the statistical properties of the real systems. Several mobility networks at different scales – intra-city [52, 53], inter-city [54, 55], country scale [55], worldwide scale [56, 57] – and of different type – air travel [56, 57], commuting [54, 55], movement of people between city locations [52, 53] – have been studied and found to exhibit large-scale heterogeneities at different levels. In particular, the number of connections from a given location (i.e. the degree k of a node) is generally described by a broad distribution $P(k)$, with $P(k)$ representing the probability that a randomly extracted node has degree k. In addition, the fluxes of traveling people (the weight w_{ij} of the link connecting i to j [56]) are also found to be characterized by very large fluctuations with a weight probability distribution $P(w)$ spanning over several orders of magnitude. Finally, a statistical law relating the travel flux w_{ij} to the number of connections departing from the two ending nodes i and j was found in the worldwide air transportation network [56]:

$$w_{ij} \sim (k_i k_j)^{\theta}. \tag{26}$$

These properties are illustrated in Fig. 3 for the case of three empirical mobility networks characterized by different spatial scales: the air transportation network analyzed in [56], the commuting patterns among counties in the United States [58] and among municipalities in Italy [59]. The figure reports for the three datasets the results for the degree distribution $P(k)$, and the travel fluxes w_{ij} as functions of the topology expressed in terms of $k_i k_j$. All networks display large heterogeneities in the degree distribution and exhibit travel fluxes consistent with Eq. (26). It is worth to note that these statistical features are invariant under changes of the mean of transportation and of the spatial scale, thus pointing out their robustness as peculiar aspects characterizing these systems.

Following the empirical analysis of Figure 3, we assume a metapopulation model whose underlying structure is heterogeneous to include degree fluctuations, and characterized by travel fluxes following Eq. (26). Both topology and travel fluxes are therefore expressed in terms of the degree k of each subpopulation. A convenient description is then provided by the degree-block variables of the metapopulation system [60], where each quantity that depends on a subpopulation i (e.g. population size, number of infectious, etc.) is defined in terms of the subpopulation degree k_i. This corresponds to a mean-field assumption for which subpopulations with a given degree k are considered statistically equivalent.

Within this theoretical framework, Eq. (1) of the main paper describes the evolution of the disease invasion at the subpopulation level in a coarse grained view in which the subpopulations are

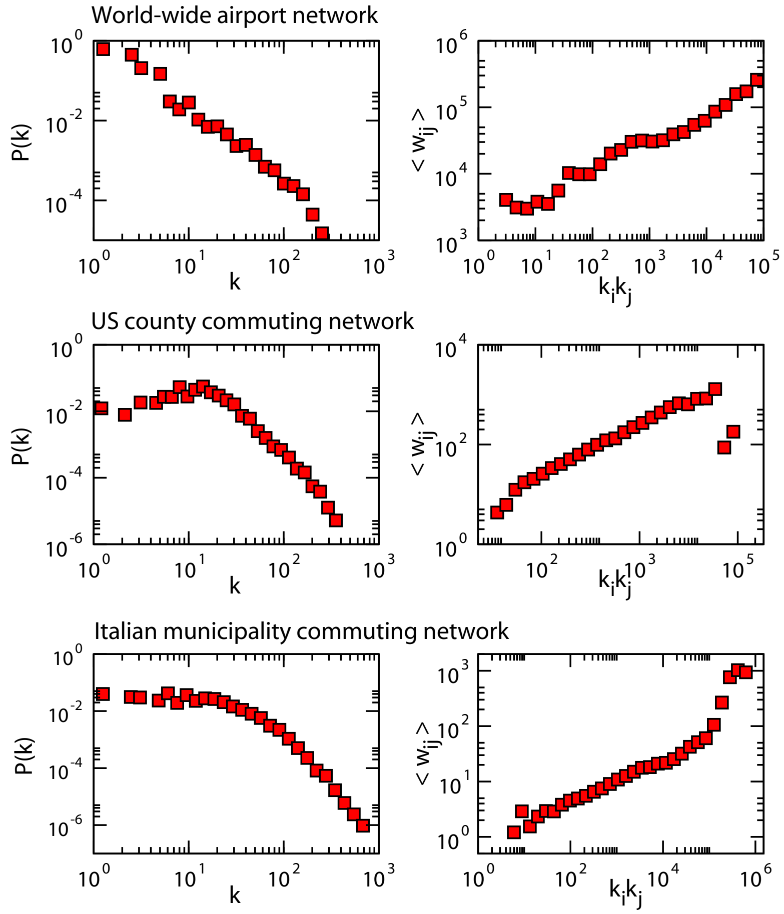

Figure 3: Degree distributions and average weight of the connections as a function of the product of connected node degrees for three empirical mobility networks.

the basic elements of the process [61, 62]. We assume that a local outbreak starts in a given subpopulation and then spreads from one subpopulation to others by means of infectious individuals traveling along the underlying mobility network. The link between the microscopic dynamics of the infection transmission among individuals and the coarse grained description at the metapopulation level is encoded in the term $\lambda_{k'k}$. It represents the number of seeds traveling from the diseased subpopulation k to the neighboring subpopulation k' during the entire duration of the outbreak, and it depends on the details of the diffusion process of individuals as well as the individual travel behavior and its interplay with the disease stages.

Following the results presented in Figure 3, we assume that the rate of diffusion on any given edge from a subpopulation of degree k to a subpopulation of degree k' is inversely proportional to the population size N_k of the origin location, and scales linearly with the travel flux $w_{kk'}$ from k to k', i.e. $d_{kk'} = \frac{w_{kk'}}{N_k} = \frac{w_0 (kk')^\theta}{N_k}$, where we used the statistical law of Eq. (26) observed in real mobility networks. In order to explicitly compute $\lambda_{kk'}$ we need to specify the compartmentalization chosen for the disease modeling. Here we extend the analysis of Ref. [61, 62] and we explore more structured and realistic compartmentalizations that take into account the presence of latent and asymptomatic individuals and envision a possible modification of the traveling behavior after presenting clinical symptoms.

More in detail, we consider the full compartmental model used in the main paper (see Fig.1 in the main paper). A susceptible individual in contact with a symptomatic or asymptomatic infectious person contracts the infection at rate β or $r_\beta \beta$, respectively, and enters the latent compartment where he is infected but not yet infectious. At the end of the latency period, denoted by ϵ^{-1}, each latent individual becomes infectious, entering the symptomatic compartments with probability $1-p_a$ or becoming asymptomatic with probability p_a. The symptomatic cases are further divided between those who are allowed to travel, with probability p_t, and those who would stop traveling when ill, with probability $1 - p_t$. Infectious individuals recover permanently with rate μ.

The number of seeds $\lambda_{kk'}$ can be approximated to the first order by

$$\begin{aligned} \lambda_{kk'} &= d_{kk'} \left[(p_t(1-p_a) + p_a) \, (\epsilon^{-1} + \mu^{-1}) \mathcal{S}_\infty N_k + (1-p_t)(1-p_a)\epsilon^{-1} \mathcal{S}_\infty N_k \right] \\ &= d_{kk'} \mathcal{S}_\infty N_k \left(\epsilon^{-1} + (p_t(1-p_a) + p_a)\mu^{-1} \right) , \end{aligned} \tag{27}$$

since each of the $\mathcal{S}_\infty N_k$ infectious individuals (with $\mathcal{S}_\infty$ being the epidemic size [34]) can travel with rate $d_{kk'}$ during a time period that is determined by his stage of disease. Asymptomatic individuals and a fraction p_t of the symptomatic can diffuse out of the diseased subpopulation during a time window that equals the sum of the average latency and infectious periods, whereas the non-traveling symptomatic individuals can only diffuse during their latency state.

Assuming an uncorrelated network so that $P(k|k') = k'P(k')/\langle k \rangle$ [64], and a disease with a reproductive ratio close to the epidemic threshold, i.e. $R_0 - 1 \ll 1$, one obtains for the global invasion the threshold condition:

$$R_* = (R_0 - 1)\mathcal{S}_\infty \left[\epsilon^{-1} + \mu^{-1} \left(p_t(1-p_a) + p_a \right) \right] w_0 \frac{\langle k^{2+2\theta} \rangle - \langle k^{1+2\theta} \rangle}{\langle k \rangle} > 1. \tag{28}$$

By explicitly introducing the expression of the epidemic size $\mathcal{S}_\infty$ for an SEIR local dynamics with

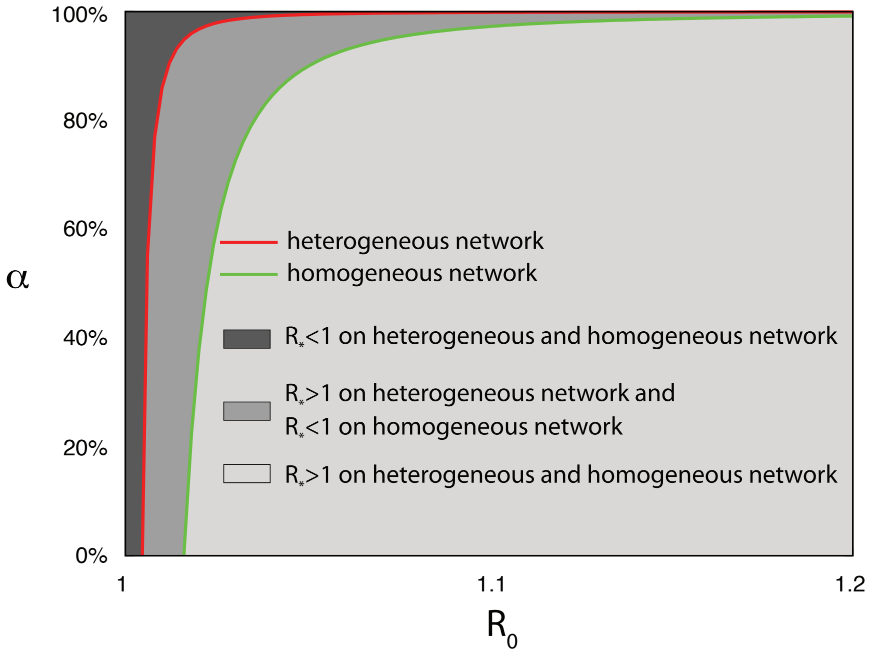

Figure 4: Two-dimensional projection of the functional $R_*(R_0, \alpha)$. Different gradations of grey distinguish the regions of the parameters space above and below the global epidemic threshold, while the red and the green curves indicate the epidemic threshold $R_*(R_0, \alpha) = 1$ for the heterogeneous an homogeneous network respectively.

R_0 close to 1 [63], we obtain the following expression for the global invasion threshold R_*:

$$R_* = \frac{2(R_0-1)^2}{R_0^2}\left[\epsilon^{-1} + \mu^{-1}\left(p_t(1-p_a)+p_a\right)\right] w_0 \frac{\langle k^{2+2\theta}\rangle - \langle k^{1+2\theta}\rangle}{\langle k\rangle}. \quad (29)$$

As described by Eq. (2) in the main paper, R_* is the product of three functions that depend on the disease parameters, as well as the topology and fluxes of the mobility of individuals. Travel-related interventions can be modeled as the reduction of the mobility scale w_0 or the reduction of the traveling probability p_t of symptomatic cases. The effect of such interventions is however damped by the large topological fluctuations of human mobility patterns, since the more heterogenous is the metapopulation network and the larger is the ratio $\langle k^{2+2\theta}\rangle/\langle k\rangle$.

In order to better understand the crucial role of the topological heterogeneity of the mobility network, we compute R_* for a homogeneous network with the same average values of degree $\langle k\rangle$ and weight $\langle w\rangle$ of the heterogeneous one. In this case, all nodes have the same degree $\langle k\rangle$ and all the links are characterized by the same weight $\langle w\rangle$, which leads to a traveling rate $d_{kk'}$ that is simply $\langle w\rangle/N$ through all the connections. Then, the number of seeds is given by $\lambda_{kk'} = \langle w\rangle \mathcal{S}_\infty \left(\epsilon^{-1} + (p_t(1-p_a)+p_a)\mu^{-1}\right)$. Replacing this term in Eq. (1) in the main paper, we obtain

$$R_* = \frac{2(R_0-1)^2}{R_0^2}\langle w\rangle\left(\langle k\rangle - 1\right)\left[\epsilon^{-1} + \mu^{-1}\left(p_t(1-p_a)+p_a\right)\right]. \quad (30)$$

Figure 4 compares the heterogeneous and homogeneous network, and shows for both cases the two-dimensional projection of the functional $R_*(R_0, \alpha)$ (α indicates the travel reduction affecting w_0 in Eq. (29) and $\langle w\rangle$ in Eq. (30), respectively). In both cases the epidemiological parameters, ϵ, μ, p_a and p_t, are set as described in Subsection 2.1. The red and green curves indicate the epidemic threshold $R_*(R_0, \alpha) = 1$ for heterogeneous and homogeneous networks, respectively. The picture highlights how the heterogeneity of the mobility network is responsible for favoring the epidemic invasion.

References

[1] International Air Transport Association. *Influenza A(H1N1) - Known measures*, http://www.iata.org/whatwedo/safety_security/safety/health_safety/measures.htm

[2] Reuters, *FACTBOX - Measures against swine flu in Europe, April 30 2009* http://www.reuters.com/article/idUSLU205194?feedType=RSS&feedName=swineFlu&virtualBrandChannel=10521

[3] Reuters, *FACTBOX - Measures in North, South America against flu, May 4 2009* http://www.reuters.com/article/idUSTRE54300I20090504?feedType=RSS&feedName=swineFlu&virtualBrandChannel=10521&pageNumber=2

[4] Reuters, *FACTBOX - Measures in Asia against deadly flu, May 4 2009* http://www.reuters.com/article/idUSSP482703?feedType=RSS&feedName=swineFlu&virtualBrandChannel=10521

[5] The San Diego Union Tribune *Tijuana-Shanghai flights to resume, January 12 2010,* http://www.signonsandiego.com/news/2010/jan/12/china-lifting-ban-flights-mexico/

[6] The Telegraph Calcutta, *Airport flu check to stop, January 23 2010,* http://www.telegraphindia.com/1100123/jsp/calcutta/story_12017040.jsp

[7] Secretaría de Comunicaciones y Transportes, *Boletín Mensual de Estadística Operacional de la Aviación Civil en México, Diciembre 2009,* http://www.sct.gob.mx/transporte-y-medicina-preventiva/aeronautica-civil/estadisticas/estadistica-aerea-vigente/

[8] Xinhua News, *First direct flight between China and Mexico to start in May, April 16 2008,* http://news.xinhuanet.com/english/2008-04/16/content_7985039.htm

[9] Reuters, *Argentina confirms first H1N1 flu case, May 7 2009,* http://www.reuters.com/article/GCA-SwineFlu/idUSTRE5468RC20090507

[10] Reuters, *Argentina lifting flu-related ban on Mexico flights, May 14 2009,* http://www.reuters.com/article/idUSTRE54D60U20090514

[11] Xinhua News, *China suspends flights from Mexico, May 2 2009,* http://news.xinhuanet.com/english/2009-05/02/content_11297034.htm

[12] Ministerio de Relaciones Exteriores de la República de Cuba, *Flights between Mexico and Cuba to Be Restored from Monday, May 29 2009* http://embacu.cubaminrex.cu/Default.aspx?tabid=12932

[13] Reuters, *Peru has its first swine flu case; bans flights, April 30 2009,* http://www.alertnet.org/thenews/newsdesk/N29406739.htm

[14] Reuters, *Peru lifts ban on Mexico flights after flu fears, May 13 2009,* http://www.reuters.com/article/AIRLIN/idUSN1343523920090514

[15] Balcan, D., Colizza, V., Gonçalves, B., Hu, H., Ramasco J.J. & Vespignani, A. Multiscale mobility networks and the large scale spreading of infectious diseases, *Proc. Natl. Acad. Sci. USA* **106**, 21484-21489 (2009).

[16] Balcan, D., Hu, H., Gonçalves, B., Bajardi, P., Poletto, C., Ramasco, J.J., Paolotti, D. , Perra, N. , Tizzoni, M., Van den Broeck, W., Colizza, V. & Vespignani, A. Seasonal transmission potential and activity peaks of the new influenza A(H1N1): a Monte Carlo likelihood analysis based on human mobility, *BMC Medicine* **7**, 45 (2009).

[17] Colizza, V., Barrat, A., Barthélemy M., Valleron, A- J., Vespignani, A., Modeling the Worldwide spread of pandemic influenza: baseline case and containment interventions, *PloS Medicine*, **4**(1): e13 (2007).

[18] Rvachev, L. A., Longini, I. M., A mathematical model for the global spread of influenza, *Mathematical Biosciences*, **75**:3-22 (1985)

[19] Grais, R. F., Hugh Ellis, J., Glass, G. E., Assessing the impact of airline travel on the geographic spread of pandemic influenza. *Eur. J. Epidemiol.*, **18**:1065-1072 (2003).

[20] Hufnagel, L., Brockmann, D., Geisel, T., Forecast and control of epidemics in a globalized world, *Proc. Natl. Acad. Sci. (USA)*, **101**:15124-15129 (2004).

[21] Cooper, B. S., Pitman, R. J., Edmunds, W. J., Gay, N., Delaying the international spread of pandemic influenza. *PloS Medicine*, **3**:e12, (2006).

[22] Epstein, J. M., Goedecke, D. M., Yu, F., Morris, R. J., Wagener, D. K., Bobashev, G. V., Controlling Pandemic Flu: The Value of International Air Travel Restrictions, *PLoS ONE*, **2**:e401 (2007).

[23] Flahault, A., Vergu, E., Coudeville, L., Grais, R., Strategies for containing a global influenza pandemic. *Vaccine*, **24**: 6751-6755 (2006).

[24] Viboud, C., Bjornstad, O., Smith, D. L., Simonsen, L., Miller, M. A., Grenfell, B. T., Synchrony, waves, and spatial hierarchies in the spread of influenza. *Science*, **312**:447-451 (2006).

[25] Flahault, A., Valleron, A-J., A, Method for assessing the global spread of HIV-1 infection based on air travel. *Math. Popul. Stud.*, **3**:1-11 (1991).

[26] Colizza, V., Barrat, A., BarthÃl'lemy, M., Vespignani, A., The role of airline transportation network in the prediction and predictability of global epidemics, *Proc. Natl. Acad. Sci. (USA)*, **103**:2015-2020 (2006).

[27] Colizza, V., Barrat, A., Barthélemy, M., Vespignani, A., Predictability and epidemic pathways in global outbreaks of infectious diseases: the SARS case study, *BMC Med*, **5**:34 (2007).

[28] Coburn, B. J., Bradley, G., Wagner, B. G., Blower, S., Modeling influenza epidemics and pandemics: insights into the future of swine flu (H1N1), *BMC Medicine*, **7**:30 (2009).

[29] Center for International Earth Science Information Network (CIESIN), Columbia University; and Centro Internacional de Agricultura Tropical (CIAT). The Gridded Population of the World Version 3 (GPWv3): Population Grids. Palisades, NY: Socioeconomic Data and Applications Center (SEDAC), Columbia University. `http://sedac.ciesin.columbia.edu/gpw`.

[30] International Air Transport Association, `http://www.iata.org`.

[31] Official Airline Guide, `http://www.oag.com/`

[32] Keeling, M. J., Rohani, P., Estimating spatial coupling in epidemiological systems: a mechanistic approach, *Ecology Letters*, **5**:20-29 (2002).

[33] Sattenspiel, L., Dietz, K., A structured epidemic model incorporating geographic mobility among regions, *Math. Biosci*, **128**:71-91 (1995).

[34] Anderson, R. M., May, R. M.. Infectious diseases in human (Oxford University Press, Oxford 1992).

[35] Longini, I. M. , Halloran, M. E. , Nizam, A. , Yang, Y., Containing pandemic influenza with antiviral agents, *Am J Epidemiol* **159**: 623-633 (2004).

[36] Fraser, C. et al, Pandemic potential of a strain of influenza A(H1N1): early findings, *Science* **324**: 1557-1561 (2009).

[37] Carrat, F. et al, Time lines of infection and disease in human influenza: a review of volunteer challenge studies, *Am J Epidemiol* **167**: 775-785 (2008).

[38] Longini, I.M. et al, Containing pandemic influenza at the source, *Science* **309**: 1083-1087 (2005).

[39] Brote de infeccion respiratoria aguda en La Gloria, Municipio de Perote, Mexico. Secretaria de Salud, Mexico, `http://portal.salud.gob.mx/contenidos/noticias/influenza/estadisticas.html`

[40] Cruz-Pacheco, G. et al, Modelling of the influenza A(H1N1)v outbreak in Mexico City, April-May 2009, with control sanitary measures, *Eurosurveillance*, **14**: 19254 (2009).

[41] CNSNews, *Closing Mexico-U.S. Border Still an Option for Fighting Swine Flu, Congresswoman Says, April 29, 2009*, `http://www.cnsnews.com/news/article/47310`.

[42] CNN, *WHO raises pandemic alert to second-highest level, April 30, 2009*, `http://edition.cnn.com/2009/HEALTH/04/29/swine.flu/index.html`.

[43] Los Angeles Times, *Swine flu: Time to close the U.S.-Mexico border?, April 28, 2009*, `http://latimesblogs.latimes.com/washington/2009/04/swine-flu-time-to-close-the-usmexico-border.html`.

[44] Tomba, G. S., Wallinga, J., A simple explanation for the low impact of border control as a countermeasure to the spread of an infectious disease, *Math. Biosci.* **214** 70 (2008).

[45] Gautreau, A., Barrat, A. & Barthelemy, M. Arrival time statistics in global disease spread, *J. Stat. Mech.* L09001 (2007).

[46] Gautreau, A., Barrat, A. & Barthelemy, M. Global disease spread: Statistics and estimation of arrival times, *J. Theo. Bio.* **251**, 509-522 (2008).

[47] Hollingsworth, T. D, Ferguson, N. M. & Anderson R. M. Will travel restrictions control the international spread of pandemic influenza?, *Nature Medicine* **12**, 497 (2006).

[48] Eichner, M., Schwehm, M., Wilson, N., Baker, M. G. , Small islands and pandemic influenza: potential benefits and limitation of travel volume reduction as a border control measure, *BMC Infectious Diseases*, **9**: 160 (2009).

[49] Goel, N.S. & Richter-Dyn, N. *Stochastic models in Biology*, (The Blackburn Press, New Jersey, 1974).

[50] Kendall, D.G. On the generalized "birth-and-death" process, *Ann. Math. Stat.* **19**, 1-15 (1948).

[51] Keeling, M.J. & Rohani, P. *Modeling infectious diseases in humans and animals*, (Princeton University Press, New Jersey, 2007).

[52] Chowell, G., *et al. Phys. Rev. E* **68**, 066102 (2003).

[53] C. L. Barrett *et al.*, Technical Report LA-UR-00-1725, Los Alamos National Laboratory (2000).

[54] A. De Montis, M. Barthélemy, A. Chessa and A. Vespignani, *Env. Planning B* doi:10.1068/b32128 (2007).

[55] R. Patuelli, A. Reggiani, S.P. Gorman, P. Nijkamp, F.-J. Bade. *Networks and Spatial Economics* **7**, 315-331 (2007).

[56] A. Barrat, M. Barthélemy, R. Pastor-Satorras and A. Vespignani, *Proc. Natl. Acad. Sci. USA* **101**, 3747 (2004).

[57] R. Guimerá, S. Mossa, A. Turtschi, L.A.N. Amaral, *Proc. Natl. Acad. Sci. USA* **102**, 7794 (2005).

[58] Bureau of Transportation Statistics (BTS), `http://www.bts.gov/`.

[59] Italian National Institute for Statistics (ISTAT), `http://www.istat.it/`.

[60] V. Colizza, R. Pastor-Satorras, A. Vespignani, *Nature Phys.* **3**, 276 (2007).

[61] V. Colizza, A. Vespignani, *Phys. Rev. Lett.* **99**, 148701 (2007).

[62] V. Colizza, A. Vespignani, *J. Theor. Biol.* **251**, 450-467 (2008).

[63] J.D. Murray. Mathematical Biology (3rd edition Berlin: Springer Verlag, 2005).

[64] S. N. Dorogovtsev and J. F. F. Mendes. Evolution of networks (Oxford University Press, Oxford 2003).
